# Effect of Hookworm Infection on Wheat Challenge in Celiac Disease – A Randomised Double-Blinded Placebo Controlled Trial

**DOI:** 10.1371/journal.pone.0017366

**Published:** 2011-03-08

**Authors:** A. James Daveson, Dianne M. Jones, Soraya Gaze, Henry McSorley, Andrew Clouston, Andrew Pascoe, Sharon Cooke, Richard Speare, Graeme A. Macdonald, Robert Anderson, James S. McCarthy, Alex Loukas, John Croese

**Affiliations:** 1 Princess Alexandra Hospital, Brisbane, Australia; 2 Queensland Institute of Medical Research, Brisbane, Australia; 3 James Cook University, Townsville, Australia; 4 Envoi Specialist Pathologists, Brisbane, Australia; 5 The Townsville Hospital, Townsville, Australia; 6 Walter and Eliza Hall Institute, Melbourne, Australia; 7 University of Queensland, Brisbane, Australia; Copenhagen University Hospital Gentofte, Denmark

## Abstract

**Background and Aims:**

The association between hygiene and prevalence of autoimmune disease has been attributed in part to enteric helminth infection. A pilot study of experimental infection with the hookworm *Necator americanus* was undertaken among a group of otherwise healthy people with celiac disease to test the potential of the helminth to suppress the immunopathology induced by gluten.

**Methods:**

In a 21-week, double-blinded, placebo-controlled study, we explored the effects of *N. americanus* infection in 20 healthy, helminth-naïve adults with celiac disease well controlled by diet. Staged cutaneous inoculations with 10 and 5 infective 3^rd^ stage hookworm larvae or placebo were performed at week-0 and -12 respectively. At week-20, a five day oral wheat challenge equivalent to 16 grams of gluten per day was undertaken. Primary outcomes included duodenal Marsh score and quantification of the immunodominant α-gliadin peptide (QE65)-specific systemic interferon-γ-producing cells by ELISpot pre- and post-wheat challenge.

**Results:**

Enteric colonisation with hookworm established in all 10 cases, resulting in transiently painful enteritis in 5. Chronic infection was asymptomatic, with no effect on hemoglobin levels. Although some duodenal eosinophilia was apparent, hookworm-infected mucosa retained a healthy appearance. In both groups, wheat challenge caused deterioration in both primary and several secondary outcomes.

**Conclusions:**

Experimental *N. americanus* infection proved to be safe and enabled testing its effect on a range of measures of the human autoimmune response. Infection imposed no obvious benefit on pathology.

**Trial Registration:**

ClinicalTrials.gov NCT00671138

## Introduction

The “hygiene hypothesis” proposes that the increasing prevalence of allergic and autoimmune diseases in developed countries may be due to the reduction in the incidence of infectious diseases [1]; epidemiologic and genetic studies suggest that the decline in the prevalence of helminth infections is a major factor [2]–[5]. Observational studies supporting this hypothesis include a report that infection with the nematode *Strongyloides stercoralis* is associated with protection against autoimmune liver disease [6], and another showing that unspecified helminth infections with peripheral eosinophilia are associated with reduced autoantigen-specific responses and disease progression in multiple sclerosis [7]. Co-infection with helminths is also known to attenuate murine models of autoimmunity and inflammatory bowel disease [8], [9].

Despite being classed as pathogens [10], helminth parasites are being proposed as treatments for allergic and autoimmune diseases [11], [12]. Preliminary observations suggest that intentional infection with the zoophilic *Trichuris suis* is safe [13], and reduces the activity of inflammatory bowel disease [13], [14]. However, dosing with *T. suis* every three weeks is required to maintain ongoing infection because the human host does not sustain development of *T. suis* to maturity [14].

*Necator americanus* (NA) is a long-lived hematophagous, human-specific (anthropophilic) gastrointestinal nematode that infects over 500 million people in developing countries where heavy infection causes iron deficiency anemia, and is associated with reduced physical and intellectual development [15]. Experimental infection of healthy volunteers with NA infective third-stage larvae (L_3_) may cause an acute, painful enteropathy [16]–[18]. Recent published data indicate that low-dose inocula of NA are better tolerated [19], and because they do not proliferate in humans, a defined dose can be administered and later fully eliminated with anthelmintic therapy [19]. A further advantage is infected individuals pose no risk to others because hookworms are soil-transmitted (geohelminths) and cannot be propagated in modern sanitary environments.

We chose NA and celiac disease (CD) to explore the relationship between helminth infection and intestinal inflammation due to a well characterised dietary antigen, gluten. Our previous studies with this hookworm have provided us with a pure source of infective larvae [18], [20]. Individuals carrying chronic infection with hookworms in endemic settings demonstrate parasite-specific TH2 responses, but TH1 and TH2 immune responses to other antigens are diminished [21].

Celiac disease is uniquely suited to explore the effects of helminth infection; it is common [22] and remission is achieved with elimination of dietary gluten allowing host-parasite interaction to be studied free of potential artefacts caused by medications. Clinical studies in volunteers with CD also have the advantage that the effects of deliberate gluten exposure can be measured by symptom response, in blood and intestinal tissue [23]–[26]. Over 90% of people with CD possess genes encoding the major histocompatability (MHC) Class II molecule, HLA-DQ2, and HLA DQ2-restricted CD4^+^ T cells specific for deamidated gluten peptides can be isolated from intestinal tissue [26]. HLA DQ2-restricted CD4^+^ TH1 cells specific for deamidated gluten including the immunodominant α-gliadin 17-mer p57-73 (QE65) peptide are also present in blood after oral wheat challenge [27].

In this study, we undertook a clinical trial to test whether NA infection reduces the immunotoxic effects of gluten in CD.

## Methods

The protocol for this trial and supporting CONSORT checklist are available as supporting information; see Checklist S1 and Protocol S1.

### Ethics Statement

The Princess Alexandra Hospital, Queensland Institute of Medical Research and Townsville Hospital Human Research Ethics Committees approved the study. Written informed consent was obtained from all subjects.

### Patients

Healthy people with CD aged 18 years or older were invited to participate through the Queensland Coeliac Society. Inclusion criteria included: 1) pre-treatment histological diagnosis of Marsh grade 3 celiac disease [25], 2) positive immunoglobulin (Ig) A anti-tissue transglutaminase (tTG) or anti-endomysial antibody, 3) HLA-DQ2 phenotype, and 4) adherence to a gluten-free diet (GFD) for at least 6 months pre-enrolment [28]. Subjects were excluded if they had 1) insulin dependent diabetes mellitus or Addison's disease; 2) life threatening allergy; 3) treatment with immunomodulatory therapies within the six months prior to enrolment (including aspirin, non-steroidal anti-inflammatory drugs, celocoxib inhibitors, statins or intramuscular and intravenous steroids); 4) an unmanaged risk of pregnancy; 5) historical, fecal or serological evidence of a prior helminth infection; 6) iron deficiency anemia, 7) any vaccination within the 30-day period prior to study commencement, or 8) an elevated level of tTG IgA.

### Study Design

This was a prospective, randomized, double-blinded, placebo-controlled trial evaluating the safety, tolerability and immunological effects of NA infection in subjects with CD in remission on gluten free diet and during wheat challenge. Participants were matched by age and gender (Table 1). Using a random number generation sequence, the researcher preparing inocula independently of the clinical support, assigned participants to the “hookworm” and “control” groups. Primary end-points included duodenal histology Marsh scores and systemic interferon-γ measured by QE65-ELISpot pre- and post-wheat challenge. Secondary end-points included a) the clinical response to inoculation with hookworm, and b) subsequent response to the *in vivo* wheat challenge, as measured by symptom and laboratory indices, intraepithelial duodenal CD4^+^, CD8^+^ and CD3^+^ lymphocyte counts (IEL), and duodenal villus height/crypt depth (Vh/Cd) ratios. Because this was a Phase 1b/2a clinical trial assessing safety, the hookworm and control sample sizes were deliberately limited to ten volunteers each. Subjects and investigators remained blinded throughout the trial.

**Table 1:** Participant demographics.

	Control Subjects (n = 10)	Hookworm Subjects (n = 10)
Age (years)^1^^,^ 2	44 (25–58)	47 (25–62)
Gender (M/F)	2/8	2/8
BMI (kg/m^2^)^3^^,^ 2	27 (22–32)	26 (18–31)
IgA tTG3 (U/ml)^4^^,^ 2	9 (5–15)	8 (5–19)
Duration (months)^5^^,^ 2	70 (10–325)	77 (15–143

1 Age at enrolment,

2 mean value (range),

3 Body Mass Index,

4 Pre-enrolment IgA anti-tissue transglutaminase (normal range below 20 U/ml),

5 duration of gluten free diet.

#### Inocula and inoculation

Inoculations were performed at wk 0 (either 10 L_3_ or placebo) and wk 12 (either 5 L_3_ or placebo). Inocula of five or ten NA L_3_ were prepared as previously described [18]. Placebo consisted of an identically presented inoculum containing 0.2 ml McIlhenny Co Tabasco Pepper Sauce® to mimic the pruritus that accompanies skin penetration by L_3_. A control sample of L_3_ was submitted with every batch of inoculum including placebo, and was inspected microscopically for larval motility to verify viability. All vials and pipettes were examined following inoculation to ensure that there were no residual hookworm larvae. Evidence of hookworm infection was provided by peripheral blood eosinophil count (×10^9^/L), and documenting hookworm eggs in feces by microscopy and/or by sighting a hookworm at endoscopy.

A strict GFD was maintained from weeks 0 to 20. Endoscopy was performed at week 20 using an Olympus high resolution digital endoscope; five single-bite biopsies were collected from the third part of the duodenum (D3), and seven from the second part (D2). Volunteers consumed two 50 g slices of white bread twice a day (equivalent to 16 g of gluten daily) for five days. On the sixth day, blood was collected and a second endoscopy was performed (referred to as the week 21 timepoint).

#### Monitoring, assessments and wheat challenge

Clinical reviews occurred at weeks 0, 4, 12, 20 and 21, and symptom diaries were recorded weekly. From weeks 0–20 and daily during the wheat challenge, participants were asked to complete a symptom score rating from 0 (none) to 3 (severe) bloating, constipation, flatulence, headaches, lethargy, mouth ulcers, nausea, skin rash, skin itch, and vomiting, and to record their number of bowel motions, loose bowel motions and urgent bowel motions, and the number of episodes of pain per day. Also recorded were pain intensity and general wellbeing on a visual analogue scale from “no pain at all” to “very severe” and “very well” to “severely ill” respectively.

### Histopathological Analysis

Biopsies were fixed in neutral buffered formalin, processed and carefully orientated and embedded in paraffin wax. Sections (3 µm) were stained with hematoxylin and eosin (H&E) and immunostained with anti-CD8, anti-CD4 and anti-CD3 antibodies (all from Novocastra Laboratories Ltd). The Marsh scores were graded by two independent researchers [25]. The IELs per 100 nucleated enterocytes (100NE) were counted at 24 randomly selected sites between the villus tip to the base of the crypt in each biopsy. The Vh/Cd ratios were measured independently of the Marsh grading [24] and were performed on 10 randomly selected well-orientated sites.

### Peripheral blood interferon-γ ELISpot responses to the immunodominant α-gliadin peptide p57–73 QE65

Peripheral blood mononuclear cells (PBMCs) were incubated with p57–73 QE65 (50 µg/ml) in overnight IFN-γ ELISpot assays, as previously described [27]. Briefly, ELISpot plates were coated with anti-IFN-γ (clone 1-D1K, Mabtech) and then blocked with 5% fetal calf serum (FCS). Freshly isolated PBMCs were added to wells containing medium alone (RPMI 1640, Invitrogen, 10% fetal bovine serum, 100 U/µl penicillin, 100 µg/ml streptomycin and 2 mM L-glutamine) or p57–73 QE65 in medium, and cultured for 24 h at 37°C in 5% CO_2_. Cells were removed and the plates were incubated with anti-IFN-γ-biotin (clone 7-B6-1, Mabtech), then streptavidin-alkaline phosphatase, and developed with BCIP/NBT substrate. Spots were counted on an iSpot ELISpot reader (Autoimmun Diagnostika GmbH).

### Statistical Analyses

Statistical analyses were performed using Prism5 (GraphPad). When comparing non-continuous variables (eg. symptom scores), area under the curve analysis from week 1 to week 20 followed by Mann-Whitney U test was used to compare between groups. For continuous variables (eg. duodenal IEL counts), two-way ANOVA was used to analyse between time-points and groups. In the figures, * = p<0.05, ** = p<0.01, *** = p<0.001.

## Results

**Figure 1:**
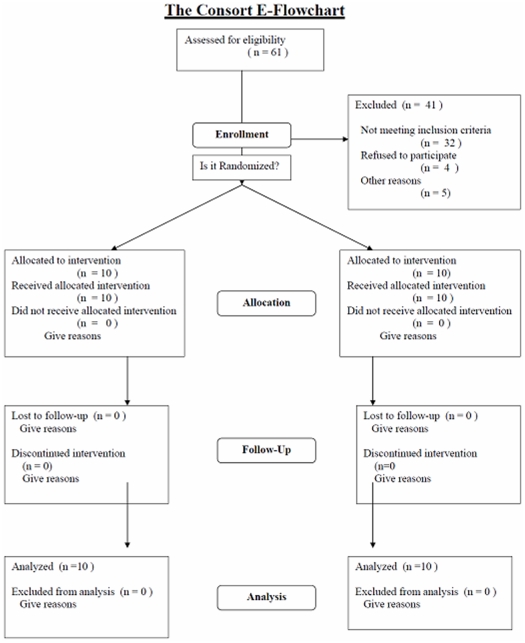
CONSORT flowchart.

**Figure 2:**
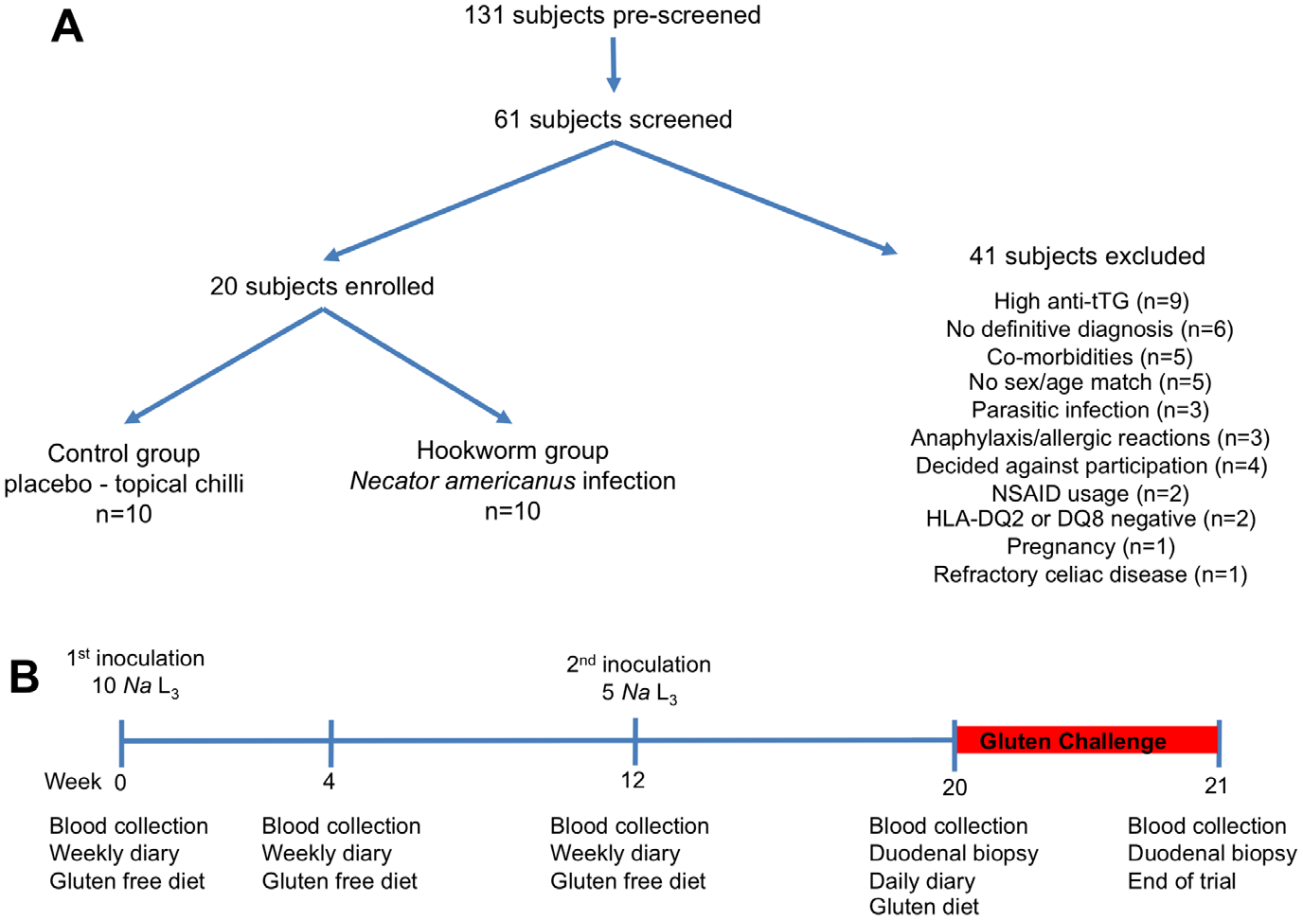
Recruitment and Protocol. Recruitment and summation of those excluded post screening (A), and trial outline (B).

Between October 2007 and March 2008, 131 potential subjects indicated interest, 61 were screened and twenty were enrolled and completed the study without protocol violations (Figure 1). Reasons for exclusion of subjects are summarised in Figure 2.

### Hookworm Tolerance and Safety

#### Inoculation efficacy

**Figure 3:**
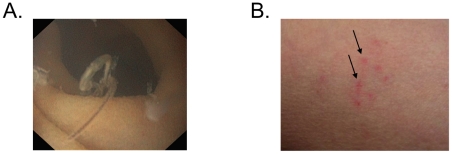
Representative photographs of parasitic infection (A) Adult hookworm (B) Inoculation site.

Mature hookworm infection was confirmed in each of the hookworm group by the identification of eggs in feces (n = 5), or by endoscopic visualisation of at least one adult worm (n = 8; Figure 3A), or by both (n = 3). In five subjects with proven infection, patency could not be confirmed by fecal microscopy despite the testing of 17 of 18 scheduled collections from wk 12–21. No control subject had evidence of hookworm infection and no subject had evidence of any other helminth infection.

#### Clinical response to *N. americanus*

**Figure 4:**
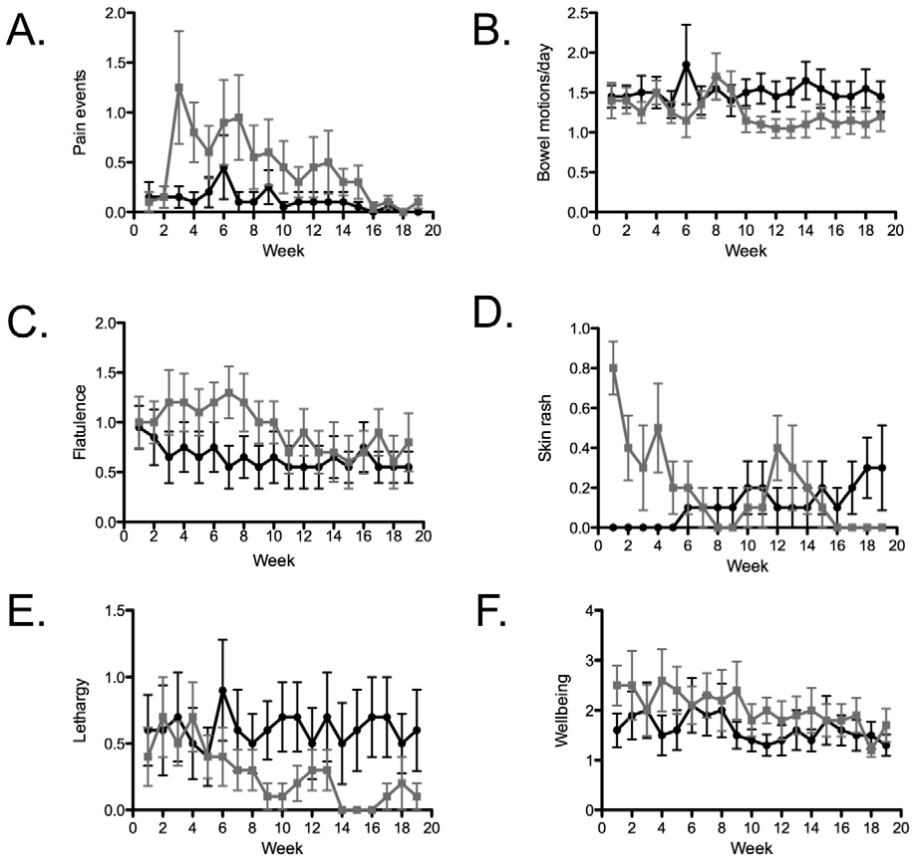
Symptom responses to hookworm infection. Control group is indicated by black circles, hookworm group by grey squares, values are mean +/− SEM. (A) pain events, (B) bowel motions/day, (C) flatulence, (D) skin rash, (E) lethargy, (F) wellbeing. Area under the curve analysis followed by Mann-Whitney U test to compare between groups showed that pain events (A) was significantly different between the groups.

*Symptom scores:* No serious adverse event relating to hookworm infection occurred. All subjects in the hookworm group developed multiple tiny papules shortly after the application of L_3_ (Figure 3B). Symptoms were scored by questionnaire over the 20 weeks of hookworm infection (Figure 4A–F). The hookworm group experienced pain during the initial colonisation of the intestine, but this resolved completely by week 16 (Figure 4A). There were trends towards the hookworm group having less frequent bowel actions after colonisation was established (Figure 4B), and more flatulence (4C), nausea and bloating (data not shown) at earlier timepoints. The inoculation site remained itchy until week 4 (Figure 4D). Unexpectedly, the hookworm group experienced less lethargy than the controls (Figure 4E), and wellbeing was unaffected (Figure 4F). At week 21, 5 of 10 subjects in the control group incorrectly guessed that they were in the active hookworm-infected group, while 8 of the 10 in the hookworm group confidently and correctly identified his or her infection status. At the end of the study all infected subjects were offered anthelminthic treatment but declined.

**Figure 5:**
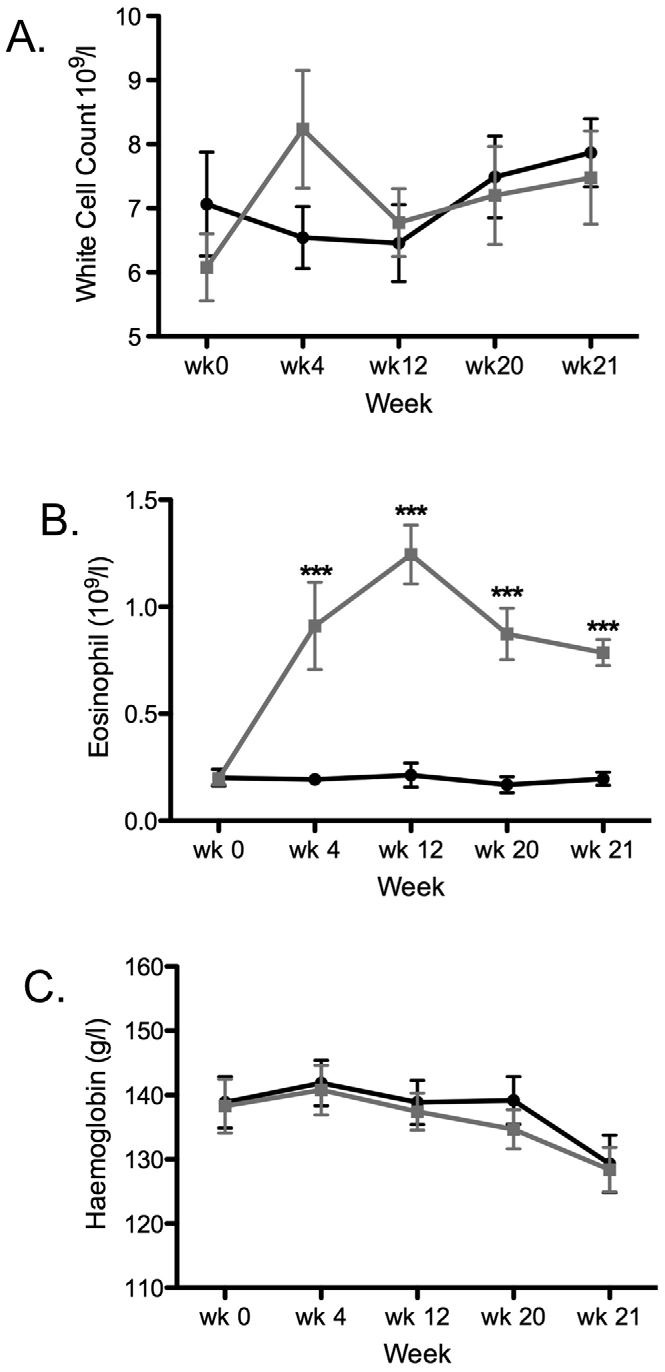
Blood cell and marker levels. (A) white cell, (B) eosinophil (C) hemoglobin. Control group is indicated by black circles, hookworm group by grey squares, values are mean +/− SEM. Data was analyzed by two-way ANOVA: significant effects of time and interaction was shown in (B), and post-hoc one-way ANOVA on each group showed the differences between timepoints as indicated.

*Laboratory Indices:* A modest leukocytosis with an eosinophilia was noticeable from week 4 after hookworm inoculation (Figure 5A–B). Hemoglobin levels were stable from week 0 to 20, but dropped at week 21 in both groups after wheat challenge (Figure 5C).

### Oral wheat challenge

#### Primary outcomes

**Figure 6:**
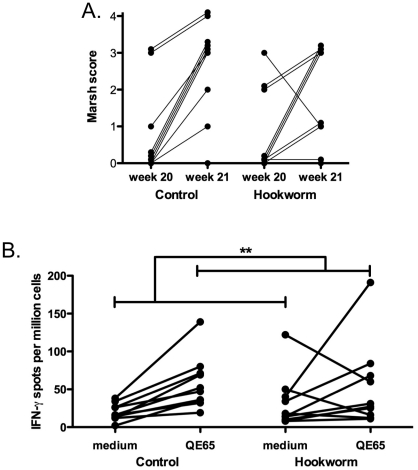
Histological and immunological results. (A) Marsh score and (B) IFN-γ ELISpot. Data was analyzed by two-way ANOVA: significant effects of time only were shown as indicated, no significant effect of group or interaction was found in (A) or (B).

*Mucosal damage measured by Marsh score:* In both the hookworm and control groups, mucosal damage deteriorated after the 5-day wheat challenge (Figure 6A). Although this deterioration was pronounced in the control group (p = 0.02), there was no statistical difference in Marsh score between hookworm and control groups.

*Systemic inflammatory immune responses:* Gluten-specific IFN-γ-producing PBMCs were measured by ELISpot before and after wheat challenge (Figure 6B). As expected, T cells secreting IFN-γ in response to p57–73 QE65 were absent prior to wheat challenge (data not shown), but were mobilised into blood following the challenge. The frequency of IFN-γ secreting T cells specific for p57–73 QE65 increased significantly only in the control group (p = 0.01), but the difference between the groups was not significant.

#### Secondary Outcomes

*Clinical response to wheat challenge — Symptom scores:* Nine subjects (4 with hookworm and 5 controls) developed abdominal discomfort, bloating and vomiting 1–2 hours after the oral wheat challenge. These symptoms resolved within 2–4 hours (data not shown). These symptoms did not recur despite continuation and completion of the five day wheat challenge. There was no significant difference between the groups during week 20 in level of pain intensity or episodes of pain, number or urgency of bowel motions, vomiting, nausea bloating, headaches, lethargy or well-being.

*Mucosal inflammatory response — Duodenal histological measurements:* After the 5-day *in vivo* wheat challenge, the total, CD3^+^ and CD8^+^ duodenal IEL counts increased in both groups (Table 2). The CD4^+^ IEL counts were not significantly affected by either *in vivo* wheat challenge or hookworm infection. A robust but almost identical decrease in the Vh∶Cd ratio after gluten was measured in the hookworm and control groups (Table 2).

**Table 2:** Duodenal cellular responses following *in vivo* gluten challenge.

	Control		Hookworm		p values		
	Week 20	Week 21	Week 20	Week 21	Time	Group	Interaction
Total IEL	30.5(14.8–46.2)	52.55(32.5–72.6)	26.4(19.4–33.4)	43.5(32.7–54.3)	**0.0037**	0.30	0.70
CD8+ IEL	32.95(19.5–46.4)	45.75(33.6–57.9)	38.55(31.8–45.3)	54.05(36.6–71.5)	**0.019**	0.24	0.82
CD4^+^ IEL	0.85(−0.043–1.74)	2.75(−0.43–5.93)	0.55(0.090–1.01)	0.7(0.087–1.31)	0.18	0.13	0.25
CD3^+^ IEL	37.65(23.4–51.9)	58.9(44.4–73.4)	42.45(34.0–50.9)	60(42–78)	**0.0039**	0.64	0.77
Villus Height	40.44(32.9–48.0)	28.23(21.8–34.6)	38.27(34.6–41.9)	28.43(19.6–37.3)	**0.0009**	0.75	0.70
Crypt Depth	15.9(14.1–17.8)	18.08(15.8–20.3)	15.38(13.5–17.3)	18.54(15.5–21.6)	**0.014**	0.98	0.64
Vh/Cd ratio	2.669(2.0–3.3)	1.644(1.2–2.1)	2.575(2.1–3.0)	1.683(1.0–2.3)	**0.0006**	0.91	0.79

Levels were analyzed by two-way ANOVA. p values considered significant are indicated in bold.

## Discussion

The “hygiene hypothesis” is a plausible explanation for the increasing incidence of autoimmune and allergic diseases in affluent societies, but it has been difficult to directly elucidate the mechanism by which infectious agents such as nematodes alter disease-causing immune responses in humans. In common with many other immune diseases, the prevalence of CD has risen dramatically. We propose that chronic helminthiasis, such as hookworm infection, may be immunomodulatory and alter pathogenic immune responses *in vivo*.

In this Phase 1b/2a trial, we have established an experimental model allowing us to explore how hookworm infection alters the effects of gluten in CD. Chronic hookworm infection can be reliably and safely established in subjects with well controlled disease. Lacking precedent, the size of this demanding study was small. We observed at best weak trends towards reduced numbers of gluten peptide-specific T cells in blood and histological damage following wheat challenge in CD.

As has been reported in another trial, standard fecal microscopy is relatively insensitive in light infection; a negative test does not exclude colonization [29]. All patients inoculated acquired adult hookworms in the intestine. The pathognomic findings of a transient papular rash at the site of inoculation, together with the common development of mild abdominal pain in the period soon after the initial inoculation hampered attempts to blind the hookworm-infected participants and investigators. In each control participant however, genuine confusion as to status was usual. It is inherently difficult to mask the hallmark features of hookworm inoculation, just as it is to mimic them. Rather than inoculating every participant and subsequently treating the controls with an anthelminthic, we felt obliged to accept this compromise. The occurrence of abdominal pain has implications when evaluating hookworms as therapy in clinical trials, especially during the establishment phase where symptom scores to measure outcomes may be confounded [20]. Because infection with NA typically persists for years [30], [31], the morbidity occurring during early infection should be accounted for by undertaking studies after chronic infection is established.

The refusal of all participants in the active arm to take anthelminthic therapy after completion of the trial was not unique to this study [20], [32] and supports the contention that chronic light hookworm infection does not compromise wellbeing, an argument congruous with the trend in the improved lethargy score. Although it is well recognized that heavy hookworm infection causes clinically significant blood loss [15], the legitimate concern that experimental infection would cause anemia in patients already predisposed with CD did not eventuate. As anticipated from a previous experimental inoculation study utilising capsule endoscopy [18], the hookworm group acquired peripheral and mucosal eosinophilia but the mucosa at week 20 was not obviously damaged.

Following the epidemiological evidence of a causal association between the disappearance of helminths from societies with advanced sanitary infrastructure and the apparent rise in incidence of autoimmune and allergic diseases [3], a number of interventional clinical trials have been undertaken [12], [20], [32], [33], with inconsistent results. The porcine whipworm, *T. suis*, has been reported as beneficial in Crohn's disease and ulcerative colitis [33], both conditions which share genetic traits with CD [34]. However, a recently reported controlled trial using this helminth in patients with allergic rhinitis demonstrated that while an immunological response to whipworm was elicited, no therapeutic benefit was apparent [12]. Similarly, in a trial where NA infection was tested for an effect among patients with asthma, no significant benefit from helminth infection was reported [32]. While our experience from a proof of concept study where patients with active Crohn's disease were infected with hookworm suggested an early benefit, their wellbeing was reliant on continuation of immunosuppressive therapies [20].

Our study establishes that hookworm infection on its own will not obviate the necessity for a restricted diet in CD. However, this experimental human challenge system appears to be a safe way of investigating the effect helminth parasites might impact on immune pathology. The advantages are that it directly addresses the human response, the disease process is not affected by the clinical imperative to use immune modulating therapy, intestinal tissue as well as blood is available for analyses and antigen stimulation testing can be effected both *in vivo* and *in vitro*.

## Supporting Information

Checklist S1 CONSORT Checklist.
(PDF)

Protocol S1 Study Protocol.
(PDF)
