# Supplementary material for: Effect of Hookworm Infection on Wheat Challenge in Celiac Disease – A Randomised Double-Blinded Placebo Controlled Trial
Source: PLoS One. 2011 Mar 8;6(3):e17366. doi: 10.1371/journal.pone.0017366 (PMC3050888; doi:10.1371/journal.pone.0017366)
Supplement: Protocol S1 — Study Protocol. (PDF) [file pone.0017366.s002.pdf]

Trial Protocol

A phase 2a, randomized, double blinded, placebo controlled, study evaluating immunity and gluten-sensitivity by inoculating Coeliac Disease patients with the human hookworm Necator americanus.
.

Version	1.2
Date:	Draft 15 February 2008
Proposed Start Date:	October 2007
Proposed end Date:	December 2008

Host Institution

Princess Alexandra Hospital
Brisbane, Australia

Sponsored by:

Queensland Institute of Medical Research
Brisbane, Australia

Funding Sponsor:
Broad Medical Research Program
The Broad Foundation
Los Angeles, California, USA

Confidentiality Statement

This document is confidential and is to be distributed fro review only to investigators, potential investigators, consultants, study staff, and applicable independent ethics committees or institutional review boards. The contents of this document shall not be disclosed to others without written authorization from PAH or QIMR (or others as applicable), unless it is necessary to obtain informed consent from potential study participants.

Team Roster

**Principal Investigator:**	Dr John Croese
Chief Investigators:	A/Prof James McCarthy<br>A/Prof Alex Loukas<br>Dr Robert Anderson
Associate Investigators:	Dr James Daveson<br>Dr Andrew Pascoe<br>A/Prof Graeme Macdonald<br>Dr Andrew Clouston<br>Dr Soroya Gaze<br>Prof Rick Speares<br>Dr Geoff Cobert
Research Nurses:	Dianne Jones<br>Sharon Cooke
Local Medical Monitor:	Dr Owen Harris
Clinical Trial Site:	Princess Alexandra Hospital<br>Ipswich Road<br>Woolloongabba 4102<br>Qld Australia
Clinical Trial Sponsor	Queensland Institute of Medical research
Funding Body	Broad Medical Research Program
	**Participating Institutional Review Boards**
**Ethical review**	**Princess Alexandra Hospital Human Research and Ethics Committee (PAH-HREC)**<br><br>**Queensland Institute of Medical research-Human Research Ethics Committee (QIMR-HREC)**

Participating Laboratories:

a. Envoi Pathology
b. Queensland Medical Laboratory
c. Queensland Institute of Medical Research

Table of Contents

Title……………………………………………………………………………………1

Team Roster………………………………………………………………………..2

Table of Contents…………………………………………………………………….3

Participant recruitment………………………………………………………………4

Pre-enrollment Assessment…………………………………………………………4

Trial Inclusion criteria………………………………………………………………...4

Trial Exclusion Criteria……………………………………………………………....5

Pre-enrolment Investigations………………………………………………………..6

Subject recruitment…………………………………………………………………..6

Trial Inoculation schedule……………………………………………………………7

Timing & Outline Clinical Visits & Study Schedule.…………………………………7

a. Pre-week 0……………………………………………………………………10
b. Week 0 ..………………………………………………………………….......10
c. Week 1- 5 ……………………………………………………………….12
d. Week 5……..…………………………………………………………........12
e. Week 6-11…………………………………………………………………12
f. Week 12……………………………………………………………………13
g. Week 13-19………………………………………………………………...13
h. Week 20……………………………………………………………………13
i. Week 21……………………………………………………………………16
j. Trial Conclusion……………………………………………………………17

Appendix A Introductory letter………………………………………………………..18

Appendix B Follow-up letter…………………………………………………………..19

Appendix C Pre-enrolment letter…………………………………………………….20

Appendix D Patient Information Letter……………..…………………………………..23

Appendix E QML Agreement…………………………………………………………….27

Appendix F Consent Form………………….…………………………………………34

Appendix G Diary card………………………………………………………………...35

Appendix H Transfer of Hookworm Protocol…………………………………………..36

Participant recruitment

Potential participants will be sourced through the Queensland Coeliac Society via letters of invitation, invited presentations or newsletter publications. (See Appendix A & B). Potential participants will be asked to contact either Dr James Daveson or Di Jones and on the basis of information supplied at that initial phone call, potentially suitable participants will be sent an appointment for a pre-enrollment interview with Dr James Daveson and RN Di Jones. (Appendix C). Based on pre-determined inclusion and exclusion criteria suitable participants will then proceed to pre-enrolment investigations, including blood tests, urine and faecal examinations.

Pre-enrolment Assessment

Candidates will be interviewed by Dr James Daveson and Di Jones at The Princess Alexandra Outpatient Department to

a) Determine eligibility according to the inclusion and exclusion criteria (see table 2a & b below)
b) Be given a detailed information letter to read and the opportunity to facilitate questions concerning the trial (Appendix D)
c) Obtain consent to obtain any further necessary details concerning their past health from doctors previously seen.

Table 2a: Trial Inclusion Criteria.

	**Inclusion criteria**
**1**	Diagnosis of coeliac disease
**1a**	Positive tTG (IgA)
**1b**	or positive anti IgA gliadin or anti-endomysial antibody test.
**1c**	Marsh score ≥3 on small bowel biopsy (subtotal villous atrophy)
**2**	Clinical, biochemical or histological improvement on gluten free diet.
**3**	Compliance with a gluten-free diet for 6 months lead-in.
**4**	Lifestyle & travel history indicative of a low risk for helminthic infection.
**5**	Good general health not on immunomodifying agents.
**6**	Ability to complete study
**6a**	Understand study & risks
**6b**	Social supports
**6c**	Workplace flexibility
**7**	Normal tTG at enrollment (<10 dependent on serology)
**8**	A HLA-DQ2 phenotype
**9**	Negative faecal test for intestinal helminthes.
**10**	Negative serological test for anti-strongyloides antibodies

Table 2b: Trial Exclusion Criteria.

	**Exclusion Criteria**
**1**	Children (age < 18)
**2**	Immunomodulating medication in 6 months pre-enrollment
**2a**	Oral or intramuscular/intravascular steroids
**2b**	Regular weekly use of aspirin
**2c**	Regular weekly use of NSAID
**2d**	Regular weekly use of COXII inhibitors
**2e**	Regular weekly use of statin medications
**3**	Clinical history indicating a likely need to use an immune suppressive agent during the course of the study.
**4**	Unmanaged risk of pregnancy
**5**	Past history of infection with helminthes (other than a past history of infection with the pinworm, Enterobius vermicularis)
**6**	History of insulin dependent diabetes mellitus or Addison’s disease
**7**	History of anaphylaxis or severe allergic reactions
**8**	Having received a vaccine within the preceding 30 days
**9**	Positive strongyloides serology
**10**	Iron deficiency anemia

Pre-enrolment urine, blood and fecal testing will be undertaken with Queensland Medical Laboratories (QML-Agreement Appendix E). Pre-enrollments investigations and costs are listed in table 4 below.

Table 4: Investigations and costs required at Pre-enrolment:

**Pre-enrolment Investigations**	**Costs**
Quantitative Anti-TTG IgA	$18.90
Total IgA	$11.10
FBC	$12.90
E/LFT	$14.85
CRP	*Cost included with E/LFT*
ESR	$6.00
IgE	$17.55
Serum tryptase	$120.00
Iron Studies	$24.85
HLA status for DQ2 & 8	$100.00
Strongyloides Serology	$22.35
HIV	*Cost included with Strongyloides serology*
HepBsAg	$22.10
Hep C Antibody	*Cost included with HepBsAg*
Vitamin A	$23.40
25(OH)2D	$32.25
Urinalysis	$15.55
Faecal Specimen-OCP	$6.80
Serum ßHCG in females of childbearing age	$21.90
**TOTAL COST PER PARTICIPANT**	**$448.60**
**TOTAL COST PER PARTICIPANT WITH ßHCG**	**$470.50**

Subject Recruitment

Two cohorts comprising twenty (20) adult subjects in total will be recruited. Prior to inoculation, they will be matched for age and gender and then randomized using a random number generation sequence. Ten (10) subjects will be enrolled into cohort A and receive two sham inoculations at weeks 0 and 12. Ten (10) will be enrolled into cohort B and be inoculated with ten (10) larvae of Necator Americanus, at week 0 and five (5) larvae at week 12. (See table 5).

Table 5: Trial Inoculation schedule:

**Table 5: Experimental Hookworm Study Schedule**		
Time **(Week)**	Control group:<br>**(n = 10)**<br><br>**Tabasco Pepper Sauce**	Cohort 2:<br>**(n = 10)**<br><br>**Hookworm**
**0**	**Sham inoculation<br>30 ml Blood**	**10 larvae<br>30 ml Blood**
**5 (4-6 window)**	**30 ml Blood for Soroya<br>FBC<br>Stool FEC**	**30 ml Blood for Soroya<br>FBC<br>Stool FEC**
**12**	**Sham inoculation<br>Stool FEC**	**5 larvae<br>Stool FEC**
**20 (18-21 window)**	**50 ml Blood<br>Stool FEC<br>Endoscopy and proctoscopy<br>Gluten challenge**	**50 ml Blood CMII<br>Stool FEC<br>Endoscopy and proctoscopy<br>Gluten challenge**
**21 (exactly 6 days post challenge)**	**End of study endoscopy<br>50 ml Blood for Soroya**	**End of study endoscopy<br>50 ml Blood for Soroya**

Participants and investigators will be blinded. Vials containing either hookworm larvae or placebo prepared in Townsville will be transported to the Princess Alexandra Hospital, where investigators will blindly inoculate participants to receive either hookworm or placebo. Prior to inoculation a “control vial” containing ten (10) larvae sent with each batch of larvae will be examined to ensure viability of the inoculums. The empty vials will then be transported to QIMR where an investigator will independently examine the vials to ensure there are no remaining hookworms.

Timing & Outline of clinical visits

Participants will be reviewed at weeks 0, 5, 12, 20 and 21. Inoculations will take place at weeks 0 and 12. Endoscopies will take place at weeks 20 and 21. (See table 6).

Table 6: Study Schedule

	Pre – enrollment Weeks		0	1-4	5	6-11	12	13-19	20	21
	Pre-enrollment Days		0				84		140	146
Obtain informed consent			×							
Screening history	×									
Complete History & Physical			×							×
Clinical Evaluation					×		×		×	
Distribute diary card			×		×		×		×	
Phone call/email (weekly) For symptom score and diary checking				×		×		×		
Collect diary card					×		×		×	×
FBC, ELFT,CRP, Tryptase, IgE (9mls)	×		×		FBC				×	×
Stool OCP/mcs	×				FEC		FEC		FEC	
Strongyloides serology	×									
MHC genotyping	×									
tTG	×									
Pregnancy test (females)	×		×				×			
HIV<br>HCV<br>HBsAg<br>(10mls)	×								×	
Iron studies	×									
Vitamin A levels	×									
$25(OH)_2D$	×									
Urinalysis	×									
βHCG (females of childbearing age)	×									
INOCULATION			×				×			
FACS (30mls)			×		×		×		×	×
Endoscopy									×	×
Proctoscopy/rectal biopsy									×	
Oral gluten challenge									×	
Rectal gluten challenge									×	
Blood volume (ml)										
Cumulative Blood Volume (ml)										

Investigations will also be performed on participants at weeks 0, 5, 20 and 21 as detailed in tables 7a, b, c & d.

Table 7a: Week 0 Investigations and costs.

**Week 0**	**Cost**
FBC	$12.90
E/LFT	$14.85
CRP	*Cost included with E/LFT*
Serum tryptase	$120.00
IgE	$17.55
5 by 1 ml aliquots of serum stored in lithium heparin tubes	*No additional cost*
**TOTAL COST PER PARTICIPANT**	**$165.30**

Table 7b: Week 5 Investigations and costs:

**Week 5**	**Cost**
FBC	$12.90
CRP	*Cost included with E/LFT*
Serum tryptase	$120.00
IgE	$17.55
5 by 1 ml aliquots of serum stored in lithium heparin tubes	*No additional cost*
**TOTAL COST PER PARTICIPANT**	**$150.45**

Table 7c: Week 5 Investigations and costs:

**Week 20**	**Cost**
FBC	$12.90
E/LFT	$14.85
CRP	*Cost included with E/LFT*
Serum tryptase	$120.00
IgE	$17.55
5 by 1 ml aliquots of serum stored in lithium heparin tubes	*No additional cost*
**TOTAL COST PER PARTICIPANT**	**$165.30**

Table 7d: Week 21 Investigations and costs:

Week 21	Cost
FBC	$12.90
ELFT	$14.85
CRP	*Cost included with E/LFT*
Serum Tryptase	$120.00
IgE-total and specific	$17.55
HepBsAg	$22.10
Hep C antibody	*Cost included with HepBsAg*
HIV Serology	$11.85
5 by 1 ml aliquots of serum stored in lithium heparin tubes<br>**TOTAL COST PER PARTICIPANT**	*No additional cost*<br>***$199.25***

Pre Week 0 Visit

1. The first shipment of hookworms from The Townsville Hospital to the Princess Alexandra Hospital will occur on the 17th March. They will be stored securely at room temperature in the Chief Scientists' Office (Dr Linda Fletcher). Inoculations will take place in the lab.
2. QML will need to be notified that four lots of blood will be collected from the Princess Alexandra Hospital at week 0.
3. Dr Soroya Gaze will need to be notified of the date so that she can be present for the collection of bloods for CMI.
4. The procedural booking nurse at the PAH will need to be made aware so that she can the endoscopies for weeks 20 and 21.
5. The RN in Endoscopy OPD will need to be notified so that she can arrange for four chairs that morning. Leanne Foxcroft will also need to be let know of the dates.
6. Each participant will be given their complete visit requirements-pending start date of inoculations.
7. Sharon Cooke will be notified of number of participants being inoculated on that day.

Week 0 (Tuesday 25 March 2008)

Participants will be inoculated at the rate of four per week, and hence "week 0" for the cohort will run over 5 weeks pending adequate recruitment of participants. The subject will present to gastroenterology outpatients at the Princess Alexandra Hospital between 8.30am and 10am. They will be notified of their appointment by Di Jones.

This visit will take roughly 1.5 hours per participant. The dressing will be left on for one hour with the participants monitored for an hour post inoculation.

At this visit,
a) RN Di Jones will:

1) Witness and countersign the consent form, (See appendix F) as will the nominated investigator.

2) Distribute a study diary. (See Appendix G). This will need to be completed on a nominated day, each week until reviewed at week 12.
3) Review contraceptive methods if applicable.
4) Ensure that bloods have been collected with QML
5) Collect an extra 50mls of blood to be given to Dr Soroya Gaze for CMI analysis.

Week 0 Investigations:

**Week 0**
FBC
E/LFT
CRP
Serum tryptase
IgE
5 by 1 ml aliquots of serum stored in lithium heparin tubes

b) Dr James Daveson will:

1) Witness and countersign the consent form, (See appendix H) as a nominated investigator.

2) Complete a history and physical examination, including an assessment of any changes to candidate medications.

c) Inoculation will occur.

1) The required number of hookworm will arrive pre-prepared at the Princess Alexandra Hospital in 0.5ml screw top tubes in 0.2ml of deionised water. They will be labeled and prepared off site by Sharon Cooke and Prof Rick Speares. Identical sham-inoculums comprising 0.2ml McIlhenny & Co Tabasco Pepper Sauce® will also arrive pre-prepared from Townsville. The site investigators will be blinded to their contents. A control vial will also arrive with each batch.

2) The 0.2ml from the control vial will be extracted from the 0.5mL screw top tube using an automatic pipette and then placed on a microscope slide under a dissecting microscope. The larvae will be checked for viability.

3) Once viability is confirmed subjects will be inoculated blindly either with 10 infective larvae or the identical sham inoculates. The 0.2ml of deionised water will be placed on an absorbent-paper dressing, applied to the skin, and placed under pressure by adhesive plastic tape and a gauze bandage for 60 minutes (Kumar and Pritchard 1992).

4) The empty tubes will be then transferred to QIMR for inspection to ensure there are no remaining larvae.

5) The forearm will be the site of inoculation, but if for some reason this is not possible, the outer thigh will be used.

6) All participants will be asked to remain in the outpatients department for an hour post inoculation.

Weeks 1-5

Either weekly phone calls by Di Jones or email to the participants will take place for symptom score and diary checking. Diary cards will be able to be returned electronically on a weekly basis. Otherwise they will be collected at the end of the 11 weeks.

Week 5(4-6)

Participants will present to the outpatient department at the Princess Alexandra Hospital. At this visit,

a) RN Di Jones will:

1) Collect the completed diary cards, not already collected via email.
2) Distribute new study diaries. This will need to be completed on a nominated day, each week until reviewed at week 12.
3) Collect a FBC, IgE and serum tryptase from each participant. Ensure that bloods have been collected with QML.
4) Collect an extra 30mls of blood to be given to Dr Soroya Gaze for Flow and Cytometric Analysis.
5) Collect a faecal sample for Faecal Egg Count (FEC) which will be transferred to Dr James McCarthy for review (Blinded)

Week 5 Investigations

**Week 5**
FBC
Serum tryptase
CRP
IgE
5 by 1 ml aliquots of serum stored in lithium heparin tubes
Faecal Egg count

Weeks 6- 11

Either weekly phone calls by Di Jones or email to the participants will take place for symptom score and diary checking. Diary cards will be able to be returned electronically on a weekly basis. Otherwise they will be collected at week 12.

Week 12

The participant will present to gastroenterology outpatients at the Princess Alexandra Hospital at a nominated appointment time.

This visit will take roughly 1.5 hours. Participants will be inoculated at the rate of four per week, and hence “week 12” for the cohort will run over 5 weeks. Inoculation will take place as per the inoculation schedule. All participants will be asked to remain in the outpatients department for an hour post inoculation.

At this visit,

a) RN Di Jones will:

1) Collect and re-distribute a study diary. This will need to be completed on a nominated day, each week until reviewed at week 20.
2) Review contraceptive methods if applicable.
3) Collect an extra 30mls of blood to be given to Dr Soroya Gaze for FACS (Flow and Cytometric Analysis).
4) Collect a faecal sample for Faecal Egg Count (FEC) which will be transferred to Dr James McCarthy for review (Blinded)
5) Perform a urinary βHCG in females of childbearing age.

b) Dr James Daveson will:

1) Complete a clinical evaluation, including an assessment of any changes to candidate medications.
2) Consent will be obtained for 2 gastroscopies with biopsy, and 1 flexible sigmoidoscopy and proctoscopy, with rectal biopsies.

Weeks 13-19

Either weekly phone calls by Di Jones or email in those patients who have expressed a desire at enrollment for this, will take place for symptom score and diary checking. Diary cards will be able to be returned electronically on a weekly basis. Otherwise they will be collected at the end of 20 weeks.

Week 20

The participant will present to gastroenterology endoscopy unit at the Princess Alexandra Hospital. This will take most of the day. Participants will need to present in the morning having fasted from midnight.

At this visit,

a) RN Di Jones will:

1) Collect and re-distribute a study diary. This will need to be completed every day during week 20.
2) Ensure that bloods have been collected.

3) Collect an extra 30mls of blood to be given to Dr Soroya Gaze for FACS (Flow and Cytometric Analysis).
4) Collect a faecal sample for Faecal Egg Count (FEC) which will be transferred to Dr James McCarthy for review (Blinded)

Week 20 Investigations:

**Week 20 QML bloods**
FBC
E/LFT
CRP
Serum tryptase
IgE
5 by 1 ml aliquots of serum stored in lithium heparin tubes

b) Dr James Daveson will:

1) Complete a clinical evaluation, including an assessment of any changes to candidate medications.
2) Supply the patient with a medical certificate for time off work if required.

ENDOSCOPIES & PROCTOSCOPIES:

Endoscopy, proctoscopy and biopsies will take place between 8.00am and 9.30am.

BIOPSY PROTOCOL-In specific order

a. From the third part of the duodenum:
    i. Two (2) passes by two (2) biopsies from D2-3 junction for immunohistochemistry (antigen stimulation testing)
    ii. Two (2) passes by two (2) biopsies from D2-3 junction for micro array analysis
    iii. Two (2) passes by two (2) biopsies from D2-3 junction for FACS
    iv. Two (2) passes for a single (1) bite each time from D2-3 junction into formalin for H&E and orientated (***must be collected last to avoid contaminating the forceps***).
    v. Two passes for two bites from D2-3 junction into formalin for H&E.

b. Examine D2 and upper jejunum for worms and ulcers. Target, collect and describe tissue where available for microarray and stimulation testing. E.g. if available,
    i. 1 pass by two (2) bites of a white spot
    ii. 1 pass by two (2) bites of a red spot

iii. Up to five passes by one bite of an immature worm
iv. Up to five passes by one bite of a mature worm

Proctoscopy:

Immediately following this while you are still sedated, a flexible sigmoidoscopy will take place. (T*he rectum should be cleared prior to the first biopsy with a saline enema*).

Rectal Wall:

a) 3 by ii large rectal wall biopsies will be taken starting at 10cm above the anus from the posterior wall.
   a. one will be for H&E in formalin
   b. one will be for immunoassays
   c. one will be for immunohistochemistry

b) 40mls of a gluten slurry (6 grams of gluten in 40mls normal saline will then be inserted (followed by 10mls of N/saline following to clear the channel).

c) 4 hours later repeat biopsies need to be taken

   a. 3 by ii "*large*" matched biopsies will be taken from the anterior wall commencing 10cm above the anus, and will be sent for:

      i. micro arrays
      ii. Immunoassays
      iii. H&E - in formalin

d) The anterior and posterior walls are to be swapped for alternating patients. (There is no need to orientate the biopsies).

e) After the biopsies, a further 200mls of N/Saline will be flushed into the rectum to clear it of remaining gluten.

f) A proctoscope, endoscope or manual technique can be employed.

All histological biopsy samples from the duodenum, jejunum and rectum will be de-identified and sent to Dr Andrew Clouston at Envoi Pathology.

GLUTEN CHALLENGE:

Once recovered, participants will be asked to undertake a gluten challenge commencing in the endoscopy unit, where they will be monitored whilst doing so. This will involve an oral gluten challenge of 2 pieces of standard white bread. Participants will then continue to consume 2 pieces of white bread twice daily for 5 days depending on tolerance. A subject who develops signs or symptoms to suggest an enteropathy will be asked to stop the challenge.

Week 21

The participant will present to the gastroenterology endoscopy unit at the Princess Alexandra Hospital at 12pm having fasted from 6am.

At this visit,

a) RN Di Jones will:
1) Collect the diary cards.
2) Review contraceptive methods if applicable.
3) Ensure that bloods have been collected by QML.
4) Collect an extra 50mls of blood in lithium heparin tubes for Dr Soroya Gaze for CMI analysis.

Week 21 investigations:

**Week 21 bloods with QML**
FBC
ELFT
CRP
Serum Tryptase
IgE-total and specific
HepBsAg
Hep C antibody
HIV Serology
5 by 1 ml aliquots of serum stored in lithium heparin tubes

b) Dr James Daveson will:
1) Complete a history and physical examination, including an assessment of any changes to candidate medications.
2) Supply the patient with a medical certificate.
3) Provide mebendazole to the participant to be taken at home.

ENDOSCOPIES:

Endoscopy and biopsies will take place between 1.00pm and 2.30pm.

BIOPSY PROTOCOL-In specific order

c. From the third part of the duodenum:
   i. Two (2) passes by two (2) biopsies from D2-3 junction for immunohistochemistry (antigen stimulation testing)
   ii. Two (2) passes by two (2) biopsies from D2-3 junction for micro array analysis
   iii. Two (2) passes by two (2) biopsies from D2-3 junction for FACS
   iv. Two (2) passes for a single (1) bite each time from D2-3 junction into formalin for H&E (***must be collected last to avoid contaminating the forceps***).

v. Two passes for two bites from D2-3 junction into formalin for H&E.

d. Examine D2 and upper jejunum for worms and ulcers. Target, collect and describe tissue where available for microarray and stimulation testing. E.g. if available,
   i. 1 pass by two (2) bites of a white spot
   ii. 1 pass by two (2) bites of a red spot
   iii. Up to five passes by one bite of an immature worm
   iv. Up to five passes by one bite of a mature worm

Up to five passes by one bite of a mature worm

All biopsy samples will be de-identified and sent to Dr Andrew Clouston at Envoi Pathology.

Trial Conclusion:

Post procedures the patients will be monitored in gastroenterology outpatients and discharged when stable as per standard procedures for the department.

Participants will be asked to take their prescribed mebendazole. Follow-up via phone call or email will take place one week post completion of the trial. Participants will be notified of de-identified results as they become available if requested.

Appendix A: Introductory letter

An important study aiming to alter gluten sensitivity

Coeliac disease, as you know better than most, is more than a nuisance. Although the diet works, no curative treatment exists. Eating out is always a gamble. We are investigating a new way to alter gluten sensitivity. We are testing if being infected with a “safe” gut parasite might allow a bit of gluten to go unnoticed.

Why parasites? For five millions years, our bodies have lived with bowel parasites. Parasites were a fact of life and we suspect they may have become an important part of the healthy mix. In modern Australia, parasites have largely disappeared. This may explain why our immune system seems to have become too reactive, and might explain why some people acquire coeliac disease. We are testing if a light infection with the human hookworm, Necator americanus, is able to restore a healthy balance to immunity.

Don’t hookworms and other parasites make people sick? Obviously yes, many infected people in undeveloped countries suffer ill health due to parasitic diseases. However, over 500 million people still have chronic hookworm infection, and most are unaware of and largely unaffected by it. Recently, we safely infected healthy adults and Crohn’s patients with hookworm. I have hookworm infection, but my wife doesn’t and she is not at risk, and I feel fine.

.

What does the study involve? The study will last 21 weeks. Participants will attend 4 appointments with the researchers at the Princess Alexandra Hospital. Appointments will be at times to suit the participants – in the evening or on the weekend if required. Parking will be arranged, and reimbursement for public transport will be available if required. Half of the participants will be given hookworm, and half will receive a dummy dose (known as a placebo). At the end of 20 weeks, everyone will undergo endoscopy and a rectal examination (when sedated) to assess what effect the worms have had. Then, everyone will be expected to eat bread for five days. If symptoms develop, the gluten can be stopped. The endoscopy will be repeated 6 days after the first, after which the hookworm infection will be treated. Hookworm infection is easily treated.

The investigators are from leading institutions throughout Australia including the Princess Alexandra Hospital and the Queensland Institute of Medical Research (QIMR).

If you would like to be part of this very important study, or simply would like more information, please call one of our team at the Princess Alexandra Hospital: Dr James Daveson 0412 089 707
Di Jones (study nurse) 0403 757 141

I look forward to your consideration.

Dr John Croese (Principal Investigator)

APPENDIX B

December
ATG 2007:

RESEARCH PROJECT
HAVE YOU HEARD ....
A clinical trial is commencing at the Princess Alexandra Hospital which is exploring a novel way of treating Coeliac Disease. Close to 30 people from the Queensland Coeliac Society who have previously indicated an interest in being involved in research are currently being enrolled in the trial. If you would like information sent to you about it or would like to speak to one of the investigators, please email Di_M_Jones@health.qld.gov.au or call 0403 757 141.

APPENDIX C: Pre-enrolment letter.

Princess Alexandra Hospital
Ipswich Road
Woolloongabba 4102

[DATE]
Dear [___________],

Thank-you for indicating your interest in participating in the trial:
Inoculating Celiac Disease patients with the human hookworm *Necator americanus*: a small study evaluating immunity and gluten-sensitivity.

A pre-enrollment appointment has been made for you at the Outpatients Department, in the Department of Gastroenterology and Hepatology at the Princess Alexandra Hospital for the:

[***Enter date & time]***

Access to the Princess Alexandra Hospital is via Ipswich Road in Woolloongabba. Free parking will be available on the basement level of the hospital if your appointment is after 5pm. You access this carpark directly from Ipswich Road. If not, parking is available in the multi-storey car park across the road from the PAH

Part of the screening programme involves assessing your suitability to be included in the trial. This includes for example simple things such as

- The likelihood of your previous exposure to helminthic (worm) infections, particularly if you have traveled extensively overseas in rural or remote areas.
- The results of prior blood tests.

We will also need to collect some blood from you at this point, as well as a faecal sample to make sure you do not currently have a hookworm infection, (this is a simple test which you can do in the privacy of your own home if you prefer).

As such, any information you have concerning your diagnosis of coeliac disease will be very useful, e.g. gastroscopy reports, blood tests or biopsy results. You should bring all of it along, as well as the items on the checklist below. We may also require the names of your general practitioner and physician or gastroenterologist to gather any other required information. A checklist is included below.

If you have any concerns or questions prior to coming to your visit, please call either Di Jones on 0403 757 141 or Dr James Daveson on 0412 089 707. We look forward to meeting you.

Yours truly,

Di Jones & James Daveson

CHECKLIST OF ITEMS FOR PRE-ENROLLMENT VISIT

1. All of your current medications (tablets)
2. Names of any additional medications you have had over the last six months.
3. Name & Address of your general practitioner
4. Name and Address of your physician or gastroenterologist.
5. Any results such as gastroscopy reports, blood tests or biopsy results concerning your diagnosis of celiac disease.

DIRECTIONS TO THE OUTPATIENT DEPARTMENT AT PAH

Gastroenterology Outpatients (also known as 4F), are located on Level 4 of the Princess Alexandra Hospital. If you are parking in the basement car park, when you arrive at the boom-gate, let security know via the intercom of your arrival. Your name will have been left with them. If there is any problem, simply have them call Dr James Daveson on 0412 089 707.

Once parked, proceed to the “ORANGE” lifts and go to level 4. (Some of the lifts will only go to the ground level, in which case you will have to transfer to another ORANGE lift to get to the 4th floor). Then simply follow the signs to the outpatient Department. If you get lost, head to ward 4E, as they will be able to direct you, or alternatively call Di Jones or James Daveson.

If you are parking in the multistory car park cross Ipswich road to get to the PAH either by the overhead walkway or via the lights, and then proceed to the ORANGE LIFTS. Again, go up to level 4 and proceed to the OUTPATIENT DEPARTMENT (4F).

To get into the multistory car park, head South along Ipswich Road. When you are virtually opposite the PA Hospital turn left into Tottenham Street, and then take the first turn left again into Wolseley Street. As you come around the corner, you will see the entrance to the multistory car park. You will be reimbursed fro the costs of parking. We do however, need a receipt for this to happen. The cost is $10 for 2 hours, and $13 for 3 hours or more.

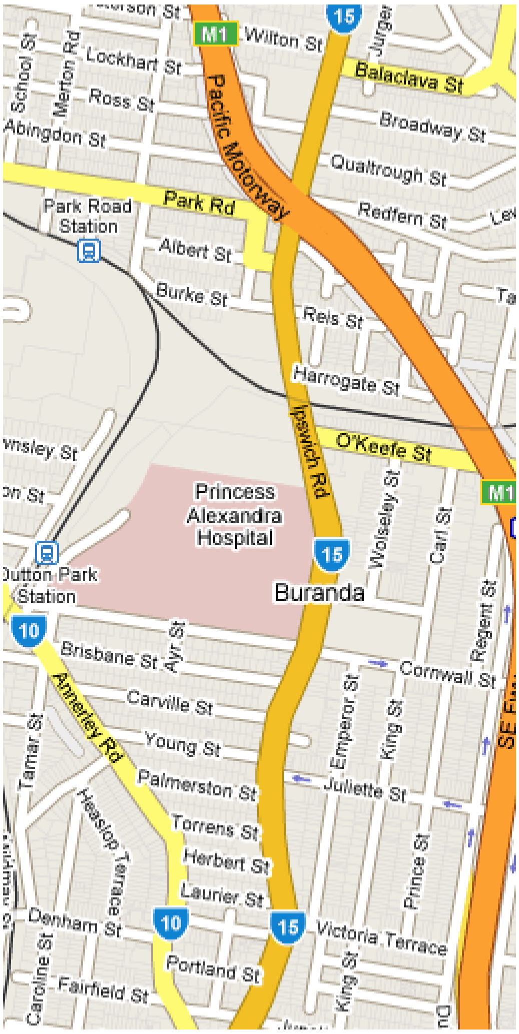
Lockhart St
Ross St
Abingdon St
Park Rd
Park Road Station
Albert St
Burke St
Pacific Motorway
Wilton St
Balaclava St
Broadway St
Qualtrough St
Redfern St
Reis St
Harrogate St
O'Keefe St
Ipswich Rd
Princess Alexandra Hospital
Buranda
Wolseley St
Carl St
Regent St
Cornwall St
Brisbane St
Ayr St
Carville St
Young St
Palmerston St
Juliette St
Emperor St
King St
Annerley Rd
Tamar St
Heaslop Terrace
Torrens St
Herbert St
Laurier St
Prince St
Victoria Terrace
Denham St
Portland St
Fairfield St
Caroline St
School St
Merton Rd
M1
15
10

Appendix D

PATIENT INFORMATION SHEET

PROTOCOL NAME: Inoculating Coeliac Disease patients with the human hookworm *Necator americanus*: a small study evaluating immunity and gluten-sensitivity.

Coeliac disease (CD) is an increasingly recognised and serious gastrointestinal illness. Both sexes and all ages are affected. No curative treatment exists, although adherence to a strict gluten-free diet is effective in most cases. Most people with coeliac disease feel vulnerable when eating outside of the home, and many have experienced relapses of symptoms after having inadvertently eaten food contaminated by gluten. We aim to test if hookworm infection can make the immune system less responsive to gluten.

We plan to inoculate 10 healthy people with CD with a human hookworm *(Necator americanus*) to test if the parasite can change immunity and reduce bowel inflammation. The immature hookworms (10 larvae initially then 5 more after 12 or 16 weeks) will be placed on the skin under a light dressing for thirty minutes. An itchy reaction that may persist for 2-3 weeks can be expected. The larvae will quickly migrate into the blood stream, pass through the lungs, get washed out in sputum into the back of the throat, swallowed and after 3-4 weeks, some will establish themselves as adults in the upper small bowel. For the next 4 weeks, an inflammatory reaction, called eosinophilic enteritis, can be expected, which may be painful and cause diarrhea, but probably won’t.

In order to rigorously test what benefit might accrue, the infected group will be compared with 10 healthy CD volunteers who will not be infected, but who will receive a sham-inoculation and undergo the same interviews and tests as the infected volunteers. The group allocation of each volunteer will be by ballot, and this will decided only after a candidate is enrolled.

Throughout the study, inoculations and assessments will co-coordinated to reduce inconvenience. You will be required to attend the Gastroenterology Clinic at The Princess Alexandra Hospital 5 times. Your study nurse will discuss any issues that might crop up, and collect your drug and health diary (a questionnaire on how you have felt). You will be encouraged to keep regular contact with your study nurse by telephone or email. Dr James Daveson, the Gastroenterology Registrar at the PA will also be available to help out at short notice should a problem arise. Blood will be collected for testing to assure that your health is not compromised, to specifically test for anaemia and to identify changes in immunity. Your family doctor will be sent a copy of the results if you wish.

Standard upper gastrointestinal endoscopy will be performed twice in each volunteer. Depending on your shape, an enteroscope (long endoscope) may be used to give better access to the small bowel. A rectal examination will also be performed immediately after the first endoscopy (when you are sedated) and a small quantity of gluten solution will be sprayed into the rectum. After 4 hours, and just before you leave the unit, biopsies will be taken from the rectum (when you are not sedated but this should not be too uncomfortable). This testing will be performed at the PA Endoscopy Unit by Dr James Daveson and a senior gastroenterologist. Sedation will be light, but you will need a responsible person to collect you from the hospital, and

you will not be able to work on the day, drive a car or perform any activity that requires your best and unaffected attention.

At the commencement of week-20, you will be required to break your diet by eating 2 pieces of standard white bread (gluten) twice a day for 3-5 days (or until you feel you might have a problem). Before starting the bread diet and immediately on completion, the most important interviews, blood collections and endoscopies will be performed. These assessments are very important as they will provide the information from which we will measure the outcomes of the study. If you feel you cannot continue eating bread for five days, you may stop and notify us of your decision. At the end of 21 weeks the participant will be given safe effective hookworm eradication therapy in the form of an oral tablet. It is important to realise that because of the lifecycle of the hookworm, and westernised sanitation the hookworms cannot ordinarily be spread to other family members.

Prior to your endoscopies you will be provided patient information sheets, and be required to formally sign consent forms to have these procedures performed. Gastroscopy, flexible sigmoidoscopy and proctoscopy are all considered safe procedures, though there are recognized risks which you will be made aware of. Everyone enrolled in this trial by the nature of the inclusion criteria will have had gastroscopies previously.

What do you need to know about *Necator americanus*? This parasite is still present throughout many countries where it is estimated to infect 500 million people. Contrary to popular opinion, human infection with hookworms only occasionally causes illness. Mild to moderate infection in a well-nourished person has little if any impact on health and mostly goes unnoticed. That is not to say, hookworm infection does not cause illness. Anaemia due to blood loss can occur, and new infections of the gut can cause abdominal pain. An itchy rash at the site of inoculation often develops and may last two weeks. Should an adverse event happen, we are confident that it can be treated. The Necator used is this study originated from Papua New Guinea. The colony was first established in an English researcher and gifted to us for our research. We have so far inoculated 11 patients with Crohn's disease and 3 doctor-researchers, and researchers in the United Kingdom are testing this same strain of Necator in their patients with allergic illnesses and Crohn's disease. All potential hookworm donors are regularly tested for HIV, hepatitis viruses and bowel pathogens. The larvae to be used in this study will be grown from eggs obtained from the faeces provided from one of our doctor-researchers. Although we do not anticipate any serious adverse consequence from being inoculated with Necator, it must be emphasised that this is experimental medicine. Our experience is limited such that we cannot assure volunteers that this study is safe.

Why use a parasite to treat CD? Firstly, you should know that the researchers have been investigating patients with Crohn's, not coeliac disease and this study is being funded by the Broad Medical Research Program – a benevolent foundation primarily focused on research that relates to inflammatory bowel disease (IBD). As well as studying if a parasite protects against gluten sensitivity, this research will investigate the pathways that operate in IBD. Successful parasites need to avoid the host's immune defenses. Mostly, they achieve this through immune regulation. Regulation is a process based on recognition and learning. It is crucial to a person's survival, with or without parasites. You may not know this, but immune cells recognize harmless as well as harmful molecules. In health, special regulatory cells recognize the harmless molecules and then spend the rest of a person's lifetime reminding the immune system not to respond. Autoimmune diseases are those illnesses that develop when the immune system recognises what should normally be a harmless

molecule, but is unable to regulate against a response. CD and IBD are different autoimmune diseases, but with many similarities. In the context of this study, we are taking advantage of being able to introduce or exclude gluten to test if a parasite might have a role in treating people with IBD as well as those with CD.

The Crohn's patients we infected felt improved and each declined treatment to expel the worms. Some of these patients are now receiving regular top-ups, but because this study is open, we are really none the wiser if the programme works. What experience have others had to justify such an unusual experiment? Researchers in the United States of America have fed IBD patients a drink containing pig whip-worms (*Trichuris suis*) and have claimed great success. Researchers at Nottingham (UK) are conducting controlled studies using hookworms in patients with allergic disease and Crohn's, and although their work is not completed, no serious adverse reaction has occurred. Have we recognised adverse events in our patients so far inoculated with Necator? One patient inoculated with 100 larvae developed persistent diarrhea. Nasty skin reactions occurred in three cases after inoculums of 25-50 larvae. We now use 5-10 larvae, and this dose has not caused any problem other than a transient itch. We are confident that the hookworms will not directly cause serious or lasting illness, but you must realise this is not a guarantee.

Why do we think parasites might work? Previously, very few individuals might have expected to get through infancy without parasites. Human immunity has molded to this certainty. We suspect people with allergic and autoimmune diseases may have an immune system that malfunctions when parasites are excluded from the balance. By infecting CD patients with hookworm, we hoped to restore a natural balance. Our experience so far has helped us develop a programme that appears safe.

CHIEF INVESTIGATOR: Dr John Croese

PARTICIPANT

DATED: ______________________________

SIGNATURE OF CONTACT INVESTIGATOR:

DATED:

____

WITNESS:

DATED:

____

Appendix E: Agreement with QML

CONFIDENTIAL

Coeliac Disease Study

James Daveson
Princess Alexandra Hospital
Ipswich Road
Woolloongabba 4102
Ph: 0412 089 707

Date: 31st January 2008

QML Pathology
11 Riverview Place
Metroplex on Gateway
Murarrie QLD 4172
PO Box 2280
Mansfield QLD 4122
Phone: (07) 3121 4515
(07) 3121 4329

<u>Re: Agreement for the provision of pathology services by QML</u>

[redacted]

Commercial Client Account Manager
QML Pathology
Tel: (07) 3121 4515
Email: Joseph.Geran@qml.com.au

Dear Mr. Geran

Provision of Testing Services for Queensland Health, Coeliac Disease Study

Thank-you for your proposal to provide pathology services for the clinical trial "A phase 2a, randomized, double blinded, placebo controlled, study evaluating immunity and gluten-sensitivity by inoculating Coeliac Disease patients with the human hookworm Necator americanus." We are pleased to be able to offer QML the contract subject to the provisions discussed below, which are all pursuant to our recent discussions and emails.

Trial Information

The trial will be conducted over a period of twenty-one weeks (per participant), and with a tentative commencement date in March, should come to a conclusion by late August/early September. Each participant will require blood collected on four separate occasions.

The **first collection** will be **pre-enrollment bloods**. Currently we expect approximately twenty-five participants (collections), all of whom will have undergone a pre-enrollment assessment. We anticipate they will all present to QML collection sites in a similar time frame for this collection once they have received their request forms.

The **second collection** will occur at **"week 0"** which we expect to take place in early March. We anticipate 4 collections per week over a 5 week period, determined by pre-enrollment eligibility and availability of patients to commence the trial. Our aim would be to have the majority of collections done over the five week period.

Every participant will then undergo their last two collections **at weeks 20** and **21**, which again we would hope to have occurred over a 6 week period for the entire cohort.

1ST COLLECTION: "PRE-ENROLLMENT INVESTIGATIONS"

Pre-enrollment	Costs
Quantitative Anti-TTG IgA	$18.90
Total IgA	$11.10
FBC	$12.90
E/LFT	$14.85
CRP	*Cost included with E/LFT*
ESR	$6.00
IgE	$17.55
Serum tryptase	$120.00
Iron Studies	$24.85
HLA status for DQ2 & 8	$100.00
Strongyloides Serology	$22.35
HIV	*Cost included with Strongyloides serology*
HepBsAg	$22.10
Hep C Antibody	*Cost included with HepBsAg*
Vitamin A	$23.40
25(OH)2D	$32.25
Urinalysis	$15.55
Faecal Specimen-OCP	$6.80
Serum ßHCG *in females of childbearing age*	$21.90
**TOTAL COST PER PARTICIPANT**	**$448.60**
TOTAL COST PER APRTICIPANT WITH ßHCG	***$470.50***

Thus, for the first collection, we will require **three (3**) forms, with the following details.

FORM 1: PRE-ENROLLMENT INVESTIGATIONS
1. FBC
2. ELFT
3. CRP
4. ESR
5. IgE
6. Serum Tryptase
7. Iron Studies
8. Strongyloides Serology
9. Vitamin A & D levels
10. Anti-TTG quantitative levels
11. Total IgA level
12. HepBsAg
13. Hepatitis C antibody
14. HIV
15. Serum βHCG (which we will cross out if not relevant)
16. HLA status for DQ2 & 8

FORM 2: PRE-ENROLLMENT INVESTIGATIONS
1. Urine analysis for mcs, protein

FORM 3: PRE-ENROLLMENT INVESTIGATIONS
2. Faecal specimen - ocp: direct smear & concentration test

The clinical information for all **Forms 1, 2 &3** would be would be:
Pre-enrollment-Coeliac disease and hookworm trail

2ND COLLECTION: "WEEK 0"

Week 0 requires the following bloods to be collected.

**Week 0**	**Cost**
FBC	$12.90
E/LFT	$14.85
CRP	*Cost included with E/LFT*
Serum tryptase	$120.00
IgE	$17.55
5 by 1 ml aliquots of serum stored in lithium heparin tubes	*No additional cost*
**TOTAL COST PER PARTICIPANT**	***$165.30***

Please note that as discussed & as above, we require an **additional 5mls of serum collected** per participant at this visit. It will need to be prepared as **5 by 1ml aliquots and stored at -70 degrees**. We are happy to supply the **1.8ml NUNC Cryotubes CAT No. 375418 (or equivalent)** containers if required. As discussed, QML is happy to facilitate the preparation and transfer of these specimens at no additional cost. Storage will take place at the **Princess Alexandra Hospital** and they need to be delivered to the following:

Central Specimen Reception
Princess Alexandra Hospital
Attention: **Dr Linda Fletcher**
Telephone 3240 2079 or 3240 2103

The request form should be prepared as follows:

FORM 4: WEEK 0 INVESTIGATIONS

1. FBC
2. ELFT
3. CRP
4. Serum Tryptase
5. IgE levels-Total and Specific
6. 5mls of serum to be prepared as 5 by 1 ml aliquots and forwarded to:
   Central Specimen Reception
   Princess Alexandra Hospital
   Attention: **Dr Linda Fletcher**
   Telephone 3240 2079 or 3240 2103

The clinical information for **REQUEST FORM 4** would be:
Week 0-Coeliac disease and hookworm trail

3RD COLLECTION: "WEEK 20"

Week 20 requires the following bloods to be collected.

**Week 20**	**Cost**
FBC	$12.90
E/LFT	$14.85
CRP	*Cost included with E/LFT*
Serum tryptase	$120.00
IgE	$17.55
5 by 1 ml aliquots of serum stored in lithium heparin tubes	*No additional cost*
**TOTAL COST PER PARTICIPANT**	***$165.30***

Please note that as discussed & as above, we require an **additional 5mls of serum collected** per participant at this visit. It will need to be prepared as **5 by 1ml aliquots and stored at -70 degrees**. We are happy to supply the **1.8ml NUNC Cryotubes CAT No. 375418 (or equivalent)** containers if required. As discussed, QML is happy to facilitate the preparation and transfer of these specimens at no additional cost. Storage will take place at the **Princess Alexandra Hospital** and they need to be delivered to the following:

Central Specimen Reception
Princess Alexandra Hospital
Attention: **Dr Linda Fletcher**
Telephone 3240 2079 or 3240 2103

The request form should be prepared as follows:

FORM 5: WEEK 20 INVESTIGATIONS

1. FBC
2. ELFT
3. CRP
4. Serum Tryptase
5. IgE levels-Total and Specific
6. 5mls of serum to be prepared as 5 by 1 ml aliquots and forwarded to:
   Central Specimen Reception
   Princess Alexandra Hospital
   Attention: **Dr Linda Fletcher**
   Telephone 3240 2079 or 3240 2103

The clinical information for **REQUEST FORM 5** would be:
Week 20-Coeliac disease and hookworm trail

4TH COLLECTION: "WEEK 21"

Week 21 requires the following bloods to be collected.

Week 21	Cost
FBC	$12.90
ELFT	$14.85
CRP	*Cost included with E/LFT*
Serum Tryptase	$120.00
IgE-total and specific	$17.55
HepBsAg	$22.10
Hep C antibody	*Cost included with HepBsAg*
HIV Serology	$11.85
5 by 1 ml aliquots of serum stored in lithium heparin tubes	*No additional cost*
**TOTAL COST PER PARTICIPANT**	***$199.25***

Please note that as discussed & as above, we require an **additional 5mls of serum collected** per participant at this visit. It will need to be prepared as **5 by 1ml aliquots and stored at -70 degrees**. We are happy to supply the **1.8ml NUNC Cryotubes CAT No. 375418 (or equivalent)** containers if required. As discussed, QML is happy to facilitate the preparation and transfer of these specimens at no additional cost. Storage will take place at the **Princess Alexandra Hospital** and they need to be delivered to the following:

Central Specimen Reception
Princess Alexandra Hospital
Attention: **Dr Linda Fletcher**
Telephone 3240 2079 or 3240 2103

The request form should be prepared as follows:
FORM 6: WEEK 21 INVESTIGATIONS

1. FBC
2. ELFT
3. CRP
4. Serum Tryptase
5. IgE levels-Total and Specific
6. HepBsAg
7. Hepatitis C Antibody
8. HIV Serology
9. 5mls of serum to be prepared as 5 by 1 ml aliquots and forwarded to:
   Central Specimen Reception
   Princess Alexandra Hospital
   Attention: **Dr Linda Fletcher**
   Telephone 3240 2079 or 3240 2103

The clinical information for **Request Form 6** would be:
Week 21-Coeliac disease and hookworm trail

COLLECTIONS	COSTS/PARTICIPANT	COSTS/COHORT
**1ST COLLECTION** Pre-enrollment ***with serum ßHCG***	$470.50	$11,771.25 (based on 25 collections)
**1st COLLECTION** Pre-enrollment ***without serum ßHCG***	$448.60	$11,215.00 (based on 25 collections)
**2nd COLLECTION** Week 0	$165.30	$3306.00 (based on 20 collections)
**3rd COLLECTION** Week 20	$165.30	$3306.00
**4TH COLLECTION** Week 21	$199.25	$3985.00
**Total per participant *with serum ßHCG***	**$1000.35**	**$22,368.25**
**Total per participant *without serum ßHCG***	**$978.45**	**$21,812.00**

Please Invoice the Princess Alexandra Hospital and forward the invoice to

Dr Linda Fletcher
Senior Scientist
Department of Gastroenterology & Hepatology
4th Floor Princess Alexandra Hospital
Woolloongabba 4102

Additional information:

Electronic access of results will need to be set up for the following:

1) Dr A James Daveson
Princess Alexandra Hospital
Ipswich Road
Woolloongabba 4102
2) RN Di Jones
Princess Alexandra Hospital
Ipswich Road
Woolloongabba 4102

Thank-you for your assistance in completing the quote and facilitating the investigations required for this trial. Should you require any further information, or have any further queries please contact me directly on 0412 089 707.

Yours truly,
Dr James Daveson
Clinical Investigator
C/O Princess Alexandra Hospital
Ipswich Road
Woolloongabba 4102

Appendix F:

CONSENT FORM
PROTOCOL NAME: Inoculating Coeliac Disease patients with the human hookworm *Necator americanus*: a small study evaluating immunity and gluten-sensitivity.

CHIEF INVESTIGATOR: Dr John Croese
1. The nature and purpose of the research project has been explained to me. I understand it, and agree to take part.

2. I have been given an Information Sheet that explains the purpose of the study, the possible benefits, and the possible risks.

3. I understand that I may or may not be infected with hookworms, and may not directly benefit from taking part in the trial.

4. I understand that at the completion of the study I will be given effective hookworm eradication treatment.

5. I understand there are inherent risks associated with having gastroscopies, flexible sigmoidoscopies and proctoscopies. I understand I will be required to sign a consent form prior to having these procedures performed.

6. I understand that, while information gained during the study may be published, I will not be identified and my personal results will remain confidential.

7. I understand that I can withdraw from the study at any stage and that it will not affect my medical care, now or in the future.

8. I have had the opportunity to discuss taking part in this investigation with a family member or friend.

NAME OF SUBJECT:

SIGNED:

DATED:

I certify that I am familiar with the trial protocol and my responsibilities. I have explained the study to the volunteer and consider that he/she understands what is involved.

SIGNATURES OF STUDY NURSE RN Di Jones & DR JAMES DAVESON

Appendix G:

Week 1

Number of bowel motions	
Number of loose / urgent bowel motions	
Pain frequency per day	
Pain intensity (grade 1 to 10)	
Skin itch	
Nausea	
Shortness of breath	

Week 20

	Day 1	Day 2	Day 3	Day 4	Day 5	Day 6	Day 7
Number of bowel motions							
Number of loose / urgent bowel motions							
Pain frequency per day							
Pain intensity (grade 1 to 10)							
Skin itch							
Nausea							
Shortness of breath							

- the number of bowel motions on that day
- the number of loose or urgent bowel motions
- the occurrence of pain and its intensity (grade 1-10)
- general well-being (grade 1-10).

Appendix H: Protocol for transfer of Hookworms from Townsville

PROTOCOL FOR SHIPMENT OF HOOKWORMS (HW) FROM THE TOWNSVILLE HOSPITAL TO THE PRINCESS ALEXANDRA HOSPITAL

The Townsville Hospital:

Lauren Skelton/Renee Lanphier from Central Specimen Reception at The Townsville Hospital (TTH) will receive the HW from Sharon Cooke (RN). They will be packaged in an esky at room temperature and sent via directly to the Princess Alexandra Hospital (PAH). (Ideally it would be best if the transfer could commence on a Monday to Wednesday, simply to ensure they are not arriving or being transferred over the weekend). The esky will be addressed as follows:

ATTENTION DR Joan Faoagali, DIRECTOR MICROBIOLOGY PAH

Additionally they will have 3 large labels attached as described below:

DO NOT REFRIGERATE!!
DO NOT OPEN
ON ARRIVAL AT PAH CSR CONTACT 3240 2388

(3240 2388 is the generic micro PAH lab number).

PAH

ROOM TEMPERATURE ONLY

At the time the esky is dispatched, 4 people should be notified via GroupWise

1) **Dr Joan Faoagali**: joan_faoagali@health.qld.gov.au
2) **Wayne Monaghan wayne_monaghan@health.qld.gov.au**
3) **James Daveson james_daveson@health.qld.gov.au**
4) **Linda Fletcher linda_fletcher@health.qld.gov.au**

Princess Alexandra Hospital

Having arrived at PAH, please notify and transfer to *Dr Linda Fletcher*-(who is the senior scientist in the Department of Gastroenterology & Hepatology at the PA hospital). Linda has arranged a safe and appropriate storage site for the HW. Her contact details are as follows:

Dr Linda Fletcher
Senior Scientist
Department of Gastroenterology & Hepatology
4th Floor Princess Alexandra Hospital
Princess Alexandra Hospital
Woolloongabba 4102
Telephone 3240 2079 or 3240 2103

In the event of any concerns during the transfer please call Dr James Daveson on 0412 089 707
